# Beauty or the Beast? A Puzzling Modification of the Clypeus and Mandibles on the Eocene Ant

**DOI:** 10.3390/insects16080794

**Published:** 2025-07-31

**Authors:** Dmitry Zharkov, Dmitry Dubovikoff, Evgeny Abakumov

**Affiliations:** Department of Applied Ecology, Faculty of Biology, St. Petersburg State University, 7/9 Universitetskaya nab, St. Petersburg 199034, Russia; d.dubovikoff@spbu.ru

**Keywords:** Formicidae, Baltic amber, µCT, crown ant, 3D model

## Abstract

Some Cretaceous ants belonging to the stem group of Formicidae exhibit bizarre morphology. This wide range of unusual adaptive features is primarily related to the mouthparts and clypeus. The researchers were perplexed by their specific ecology, as modern ant lineages do not exhibit anything similar. Here, we report and describe a new genus based on an extraordinary and mysterious alate ant from Late Eocene Baltic amber. Undoubtedly, the new ant is classified within the subfamily Formicinae (one of the crown groups), yet it displays a highly specialised morphology and an unusual array of features that are not observed in any extant ant lineages. Neither recent nor extinct ants have such a combination of features. While the exact phylogenetic placement of the new ant remains uncertain, we offer a discussion of its potential affinities based on our constrained phylogenetic analyses. We propose that †*Eridanomyrma* gen. n. should be considered in the new tribe †Eridanomyrmini trib. n. This new taxon highlights the adaptive diversity of a highly specialised, extinct lineage of Eocene crown-group ants. We also present a 3D model based on X-ray computed microtomography (µCT).

## Simple Summary

Ants (Hymenoptera: Formicidae) represent one of the most ecologically dominant animal groups today, yet their evolutionary trajectory has been shaped by dramatic shifts in morphology and ecology over tens of millions of years. While stem-group ants from the Cretaceous have revealed bizarre adaptations—such as cranial horns, these features were thought to be largely absent in crown-group ants, particularly those appearing after the Cretaceous–Paleogene boundary. Prior to this study, no crown-group of fossil ants had exhibited such highly specialised structures, which are reminiscent of extinct stem taxa but linked to modern lineages. Our discovery bridges the gap between stem-group innovations and crown-group diversification, challenging existing paradigms about the adaptative potential of crown ants.

## 1. Introduction

Ants (Hymenoptera: Formicidae) are one of the most ecologically dominant groups of animals on the planet [1,2]. Moreover, their ecological dominance is not limited to the present, and the rich palaeontological record of ants suggests that they have been involved in symbioses and ecological interactions for tens of millions of years. The earliest reliable fossils of Formicidae were found in Charentese Amber in France (~100 mya) [3,4,5] and in Burmese amber (99 mya) [6,7,8,9,10,11,12,13,14,15,16,17,18,19,20]. Representatives of the crown subfamilies already appeared in the Cretaceous [21,22]. However, the major taxonomic and morphological diversity was represented by extinct subfamilies [23]. The rarity of their fossils (about 1% of all insect fossils) suggests that they were relatively insignificant components of Mesozoic ecosystems [23,24,25]. It is evident that these stem ants existed in palaeoecosystems until the conclusion of the Mesozoic era, at which point they underwent their final extinction, presumably at the Cretaceous–Paleogene boundary [26]. During the Cenozoic era, there have been discernible alterations in the composition and abundance of ant fossils. Nevertheless, the number of Paleocene fossil insect localities is small [27]. In this regard, the Eocene epoch is of particular significance in the context of the evolution of modern myrmecofauna, as it is during this period that there was a substantial and significant increase in the number of ant fossils. In certain Eocene sediments, they have been found to account for up to 20% of the insect present [28]. Apparently, it was at this time that they achieved the significant ecological dominance that we are witnessing now. Some ants of the stem group exhibit a wide range of unusual adaptive features, primarily related to the mouthparts [1,2,3]. Among these features are horns originating from the extensions of the clypeus. The cranial horns present in †*Aquilomyrmex* Perrichot et al., 2020, †*Chonidris* Perrichot et al., 2020, and †*Dhagnathos* Perrichot et al., 2020, are the product of an anterior clypeal margin [10]. Such cranial features display a series of morphological syndromes not seen in any modern lineages.

In the present study, a new genus is described: †*Eridanomyrma* gen. nov. This extraordinary and enigmatic alate female of ant was discovered in Late Eocene Baltic amber. It exhibits a highly specialised morphology and an unusual array of features that are not observed in contemporary ant lineages. The position of the fossil is therefore subjected to phylogenetic analyses, and the results are discussed in the context of the current classification. In addition, a 3D model based on X-ray computed microtomography (µCT) is presented.

## 2. Materials and Methods

The studied specimen of fossil ant originated from the Baltic amber, Priabonian age (Late Eocene, 37.8–33.9 Ma) [29], the Prussian Formation, on the Sambia Peninsula near Kaliningrad, Kaliningrad region, Russia. The holotype of the new species is kept in the collection of the Paleontological Museum of St Petersburg State University (PMPSU), Saint Petersburg, Russia.

### 2.1. Fossil Imaging

Photography and morphological analysis of the sample were performed using a Leica M205C motorised stereomicroscope. Subsequent image processing was carried out using the Helicon Focus Pro 8, Kritta 5.0.2 and Inkscape 1.4 software. For a clearer graphical representation of the information, we created a reconstruction of a new species in the form of a straightened three-dimensional (3D) model. An array of microtomographic sections (File S4) were obtained using a NeoScan N80 High Resolution Microtomograph. Visualisation, volume rendering, and segmentation of tomographic sections were performed in 3DSlicer 5.7.0 (https://www.slicer.org/ (accessed on 20 June 2025)). The holotype of the new species was scanned with the following parameters: voltage 45 kV, current 190 µA, without a filter, with a pixel size of 1.8 microns and a resolution of 2016 × 3936 pixels per slice with a continuous 360° rotation and a camera exposure of 145 ms per frame (5577 X-ray projections). The research was carried out on the equipment of the Research Park of St. Petersburg State University (“Centre for Molecular and Cell Technologies”, project No. 109-34813, “Resource Centre for Microscopy and Microanalysis”, project No. 112-23465 and “Centre for X-ray Diffraction Studies”, project No. 103-23769). Subsequent image processing was carried out using the Helicon Focus Pro 8 (Helicon soft, Kharkiv, Ukraine) and Inkscape 1.2 (https://inkscape.org/ru/ (accessed on 20 June 2025)) software. The results of segmentation (in 3DSlicer) in the file format STL were imported into Zbrush 2022.0.6 (Maxon, Bad Homburg, Germany) for retopology and sculpting of the model. Next, Blender v4.3 (Blender Foundation, Amsterdam, The Netherlands) software was used for visualisation and animation of the ant reconstruction. The measurements were performed by volume rendering of the sample in 3DSlicer which made all morphological structures available for study (as opposed to studying samples directly in amber with the help of a microscope) and made it possible to measure with precision of 0.01 mm.

### 2.2. Description and Measurement

The dimensional values of morphological structures are given in millimetres. The following designations are used in the text:

HL—head length, maximum length of the head, measured from the transverse line connecting the posterior-most points of the occipital corners of the head to the anterior-most extremity of the clypeus (excluding horns);
HW—head width, measured along the lower line of the eyes;
SL—the maximum straight-line length of the scape measured from antennal bulb to the apex;
PdL—pedicellum length;
FI1—the length of the first flagellomere;
FI2—the length of the second flagellomere;
AnL—the maximum straight-line length of the right antenna measured from the base of the scape to the apex of the antenna;
OL—the maximum length of the right eye measured by maximum diameter;
MdL—the length of the mandible, measured from the mandibular apex to the anterior clypeus margin (excluding horns);
WL—Weber’s length: the diagonal length of the mesosoma in profile from the point at which the pronotum meets the cervical shield to the posterior basal angle of the metapleuron;
ML—mesosoma length: diagonal length of the mesosoma as measured from the anteriormost pronotal point to the posterior-most apex of the propodeal projection in dorsal view;
FwL—the length of the right forewing measured by maximum diameter;
PnL—the maximum length of the pronotum in dorsal view;
PnW—the maximum width of the pronotum in dorsal view;
MtL—mesoscutum length. Maximum length of the mesoscutum in dorsal view;
MtW—mesoscutum width. Maximum width of the mesoscutum in dorsal view;
MsL—mesoscutellum length. Maximum length of the mesoscutellum in dorsal view;
MsW—mesoscutellum width. Maximum width of the mesoscutellum in dorsal view;
PrdL—the maximum length of the propodeum in dorsal view;
PrdW—the maximum width of the propodeum in dorsal view;
PrdH—the height of the propodeum in profile, measured as the perpendicular distance from the ventral edge to the highest point of the propodeum;
PtL—the length of the petiolar node in profile, measured as the distance from the place of attachment to the propodeum to the place of attachment to the gaster;
PtW—the maximum width of the petiolar node in dorsal view;
PtH—the height of the petiolar node in profile, measured as the perpendicular distance from the ventral edge to the highest point of the petiolar node;
HFL—the maximum length of the right hind femur, measured in anterior view;
HTL—the maximum length of the right hind tibia, measured in anterior view;
GL—the length of the gaster, measured as the distance from the place of attachment of the postpetiole to the top of the gaster in ventral view;
TL—the total length of the ant (=HL + MdL + WL + PtL + GL).
Indices:
CI (cephalic index) = HL/HW;
SI1 (scape length index) = SL/HL;
SI2 (scape width index) = SL/HW;
OI1 (eye length index) = OL/HL;
OI2 (eye width index) = OL/HW;
PI1 (petiole height index) = PtL/PtH;
PI2 (petiole width index) = PtL/PtW;
MI (mesosomal index) = WL/PnW;
PRI (propodeal index) = PrdL/PrdH.
The nomenclature of the veins of the wings follows that of Perfilieva K. S. [30].

### 2.3. Phylogenetic Analyses

As shown in previous studies [31,32,33], the phylogenetic relationships within the subfamily Formicinae cannot be properly resolved based on morphological information alone (i.e., unconstrained morphology-based analyses).

Thus, to evaluate the systematic placement of the new fossil genus, we conducted constrained morphology-based phylogenetic analyses under maximum parsimony. The use of molecular-based constraints would allow a more realistic estimation of the states at ancestral nodes, and therefore contribute to a more authentic placement of the fossil [34,35,36]. The full matrix includes 50 characters (Appendix A) among which we coded 45 characters for the new fossil (File S1). Some of the characters were derived from prior studies [33,37,38] while others were interpreted de novo from comparative morphological study across the Formicinae. Each character is treated as a state which is either observed to be true (1) or false (0) for a given specimen. The morphological characteristics were evaluated from type specimens and other material imaged on AntWeb (http://antweb.org (accessed on 20 June 2025)). The constraining backbone tree was created based on trees [31,32,33].

The parsimony analyses were performed under both equal and implied weights, using R 4.1.068 and the R package TreeSearch 1.3.158 [39]. The concavity constant in the weighted analyses was set to 12, following the suggestion by Goloboff et al. [40] and Smith [41].

In the first analysis (File S3), all taxa in the morphological matrix were included. For taxa with both morphological and molecular data, their interrelationships were fixed as the backbone tree. The fossil genera (†*Eridanomyrma* gen. n. and †*Drymomyrmex* Wheeler, 1915) and other extant taxa without molecular data (*Bregmatomyrma* Wheeler, 1929) were allowed to move freely across the backbone tree [36,42,43]. The resulting tree was graphically edited with Inkscape 1.4.

In the second analysis (File S3), only the taxa of Formicinae present in the backbone tree and the new fossil genus were included. Only the new fossil ant was allowed to move freely across the backbone tree. In order to perceive the uncertainty of the fossil placement, the parsimony scores of the trees with alternative placements of the fossil were mapped to the corresponding branches of the backbone tree based on [31,32,33]. The results were visualised with the R package ggtree 6.5.260 [44] and graphically edited with Inkscape 1.4.

## 3. Results

### 3.1. Systematic Palaeontology

Class Insecta Linnaeus, 1758.

Order Hymenoptera Linnaeus, 1758.

Family Formicidae Latreille, 1809.

Subfamily Formicinae Latreille, 1809.

Tribe †Eridanomyrmini, Dubovikoff & Zharkov **trib. n.**

**Figure 1:**
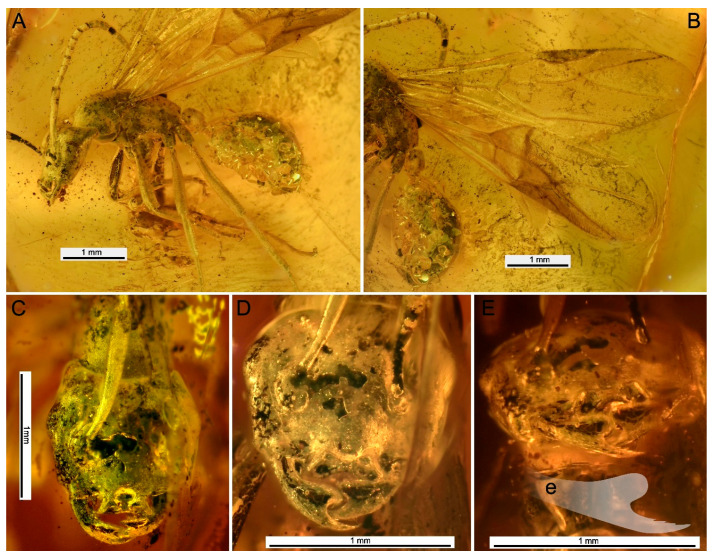
Photomicrographs of the †*Eridanomyrma unipetropolitana* sp. nov. (Hymenoptera, Formicidae) from late Eocene Baltic amber (**A**) Holotype (winged female), left lateral view. (**B**) Forewing, left lateral view. (**C**) Head, frontal view. (**D**) Clypeus and mandibles, frontal view. (**E**) Clypeus and mandibles, anterial view. (**E**) Mandible drawing (reconstruction).

**Figure 2:**
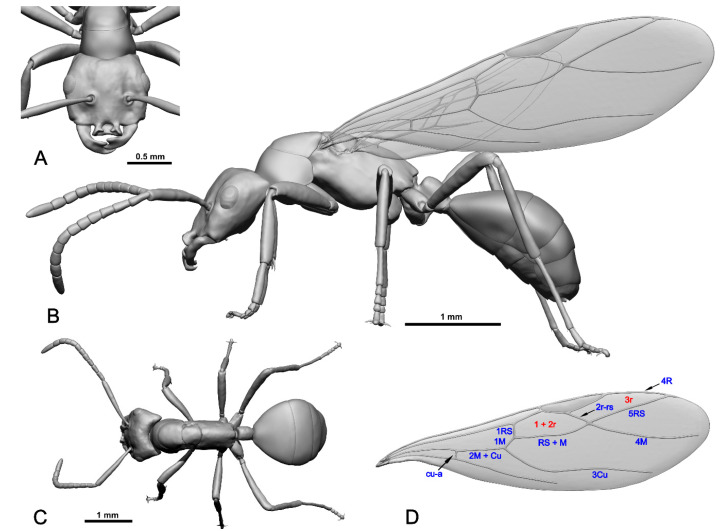
Palaeontological reconstruction (3D model) of the †*Eridanomyrma unipetropolitana* sp. nov. (Hymenoptera, Formicidae) based on X-ray computed microtomography (µCT). (**A**) Head; frontal view. (**B**) Habitus; left lateral view. (**C**) Habitus; dorsal view. (**D**) Nomenclature of the forewing cells and veins.

Genus †*Eridanomyrma*, Dubovikoff & Zharkov **gen. n.** (Figure 1 and Figure 2; Supplementary Materials Files S2 and S4).

Diagnosis for tribe: Recognisable as Formicinae and †Eridanomyrmini **trib. n.** as defined below. As for the genus, by monotypy.

Type genus: †*Eridanomyrma* Dubovikoff & Zharkov, **gen. n.** (Figure 1 and Figure 2; Supplementary Materials Files S2 and S4).

Diagnosis for genus: Winged female of †*E. unipetropolitana* **sp. n.** is distinguished from other genera of Formicinae by the following combination of characters: (1) the unique structure of the clypeus, where two protuberances of the anterior clypeal margin form distinct symmetrical double horns that point in different directions; (2) the absence of ocelli (we know of no alate females from the subfamily Formicinae that would be completely ocelli-free); (3) head flattened, with well-developed occipital corners; (4) mandibles cup-shaped, sphecoid-like, with one apical and one basal tooth; (5) petiole elongate, with long, low node.

Etymology: The generic name comes from the root *Eridano*-, which is derived from Eridanus, a hypothetical river thought to have flowed in the Eocene on the site of the modern Baltic Sea. The second part of the name, -*myrma*, is derived from the Greek *myrmex*, meaning “ant”. The gender of the name is feminine.

Type species: †*Eridanomyrma unipetropolitana* Dubovikoff & Zharkov, **sp. n.** (Figure 1 and Figure 2; Supplementary Materials Files S2 and S4).

Type material: Holotype: alate female ♀, deposited in PMPSU, Paleontological Museum of St Petersburg State University, Saint Petersburg, Russia. The specimen is located in a piece of amber: Length 11.5 mm, width 8 mm, maximum height 8 mm, minimum height 4 mm. A complete specimen observed in profile oriented to the right. In the profile on the left it is not completely visible, since it is covered with films and bubbles. In dorsal views observed with distortion. In ventral views are not visible. In the same piece of amber, the remains of larvae of Acari: Parasitengona were found, as well as numerous dark bubbles, films and debris.

Type stratum: Late Eocene, Priabonian age (37.8–33.9 Mya) [29].

Type locality: Russia: Kaliningrad Region, Baltic Sea coast, Sambia (Samland) Peninsula, Yantarny (formerly Palmnicken).

Measurements (mm): HL 1.16; HW 1.09; SL 1.14; PdL 0.19; FI1 0.18; FI2 0.16; AnL *3.14*; OL 0.29; MdL 0.45; WL 1.83; ML 1.88; FwL *4.92*; PnL 0.48; PnW 0.76; MtL 0.63; MtW 0.56; MsL 0.37; MsW 0.35; PrdL 0.76; PrdW 0.60; PrdH 0.78; PtL 0.40; PtW 0.31; PtH 0.35; HFL 1.54; HTL 1.27; GL 1.75; TL 5.59.

Indices: CI 1.06; SI1 0.98; SI2 1.05; OI1 0.25; OI2 0.27; PI1 1.14; PI2 1.29; MI 2.41; PRI 0.97.

ZooBank LSID.urn:lsid:zoobank.org:pub:065D6B7B-FD4A-432D-A834-FB719F37E1BF

Description: Alate female. *Head*. Head asymmetrical, flattened, longer than wider (CI 1.06), with well-developed occipital corners. Left occipital corner much larger than the right one. Mandibles two-pronged, cup-shaped*,* bowed, and bear small striped sculpture. Apical tooth large, sharply pointed, slightly curved inwards. Dorsal surface at base of apical tooth with small file of 3 min teeth, directed distally. Basal tooth very broad, forming rather a basal lobe. Basal margin smooth. Palps concealed basally by labrum, so that the total number of maxillary palpomeres is three or four, and the labial palpomeres are three. Maxillary palpomeres elongate. Clypeus with pair of symmetrical two-lobed protrusions, forming exceptional horns, surface finely wrinkled. Neither visually nor by computed microtomography can we say unequivocally about the nature of the structure of the protruding part of the clypeus under the horns (maybe the lower part between the horns is a film?). Frontal carinae vestigial or absent (not visible?). Right eye medium-sized, weakly bulging, above midline, with small facets. Left eye hidden by an appressed film. Ocelli absent.

*Antennae*. Antennal sockets and torular lobes exposed, the antennal fossae clearly visible. Antennae 12-merous, clubbed. Antennal scape long, strongly surpassing occipital margin (SI1 0.98). First flagellomere (FI1 0.18) approximately equal to pedicel (PdL 0.19). Following flagellomeres gradually thickening and shortening toward apex, except last two. Apical flagellomere elongate, about twice as long as penultimate. Antennas with small appressed pilosity. Setae at apex of each antennomere longer than appressed pilosity.

*Mesosoma*. Mesosoma elongate, approximately 2.4× longer than maximum height. Pronotum flat, sloping upward to mesonotum. Mesonotum angled just behind promesonotal suture. Promesonotal suture distinct, visible in dorsal view. Mesoscutum about as wide as long. Tegula concealed by bubbles and film. Axillae large. Scutoscutellar sulcus distinct. Mesoscutellum slightly longer than wide, posterior margin arched. Propodeum strongly elongated, without teeth or tubercles, dorsal surface much longer than declivous. Propodeal spiracles rounded, small, opening backwards. Metapleural gland orifice not observed (hidden under the film?). Ventral surface of mesosoma hidden by bubbles.

*Metasoma*. Petiole (abdominal segment II) elongate, finely shagreened, with long, low node. Ventral surface smooth, bearing three long setae. Gaster long, narrow, elliptical in shape. Anterior portion of abdominal segment III narrow, gradually widening to maximum width at mid-length of segment IV, then tapering posteriorly. Surface of gaster with long, erect hairs, spacing between hairs approximately equal to hair length. Distal margin of each gastral tergite with row of dense, decumbent to suberect hairs. Acidopore not visible.

*Legs*. Procoxa ~1.7× longer than maximum width, apically tapering. Profemur ~5.3× longer than maximum width, apically widening. Protibia ~5.5× longer than maximum width. Calcar thin, curved, without distinct brush; surrounded by abundant long pilosity. Probasitarsal notch gently concave, without adjacent chaetae. Probasitarsus ~1.84× shorter metatibia. Mesocoxa slightly longer than wider. Mesofemur ~6.8× longer than maximum width, apically widening. Mesotibia ~6.3× longer than maximum width. Mesobasitarsus ~1.67× shorter mesotibia. Metacoxa long, ~2.0× longer than maximum width. Metafemur ~8.3× longer than maximum width, apically widening. Metatibia ~9.7× longer than maximum width. Metabasitarsus elongate, ~1.25× shorter than metatibia. Legs covered with dense appressed pilosity. Tibiae and tarsomeres each with peg-like chaeta on posterior surface. A pair of spike-shaped chaetae located laterally on each side at apical end of tarsomers.

*Wing venation*. Forewing with closed cells 1+2r and 3r, cells rm and mcu absent. Pterostigma elongate, medium-sized, 4× longer than maximum width. Cell 3r approximately 1.4× longer than 1+2r. Vein 1RS approximately equal in length to vein 1M, both forming an obtuse angle. Vein cu-a approximately 4.5× shorter than 2M + Cu. Vein 3Cu not reaching forewing margin slightly. RS+M vein S-shaped, distinctly bent. Vein 2R-RS arising at midlength of pterostigma, directed posterodistally toward lower margin. Vein 4M departs from cell 1+2r distally from vein 2R-RS. Vein 2R-RS smoothly transitions into vein 4M, so vein 4M starts where vein 2R-RS ends. Vein 5RS nearly straight, distal end attached to 4R without curvature, forming an acute angle. Hindwing venation not evaluated due to lack of appropriate preserved views.

Males and workers are unknown.

Syninclusions: In the same piece of amber, the remains of larvae of Acari: Parasitengona were found.

Etymology: We dedicate this work to the 300th anniversary of Saint Petersburg State University, and name the species in honour of our alma mater.

Comments: An adjacent film is found throughout the ant’s body, which, for example, covers the left eye. The bubbles (near the basal part of the scape, between the forelegs, near the basal part of the wings, etc.) completely merge with the body and colour and structure, so it is not always possible to determine where the real structure of the body is and where the film is. Perhaps some seams are hidden under this film and the lower protruding part of the clypeus between the horns is also a film.

### 3.2. Phylogenetic Analyses

As demonstrated in preceding studies [31,32,33], the phylogenetic relationships within the subfamily Formicinae cannot be accurately determined solely on the basis of morphological characteristics (i.e., unconstrained morphology-based analyses). Consequently, in order to evaluate the systematic placement of the new fossil genus, constrained morphology-based phylogenetic analyses under maximum parsimony were conducted. The use of molecular-based constraints would allow a more realistic estimation of the states at ancestral nodes, and therefore contribute to a more authentic placement of the fossil [34,35,36].

**Figure 3:**
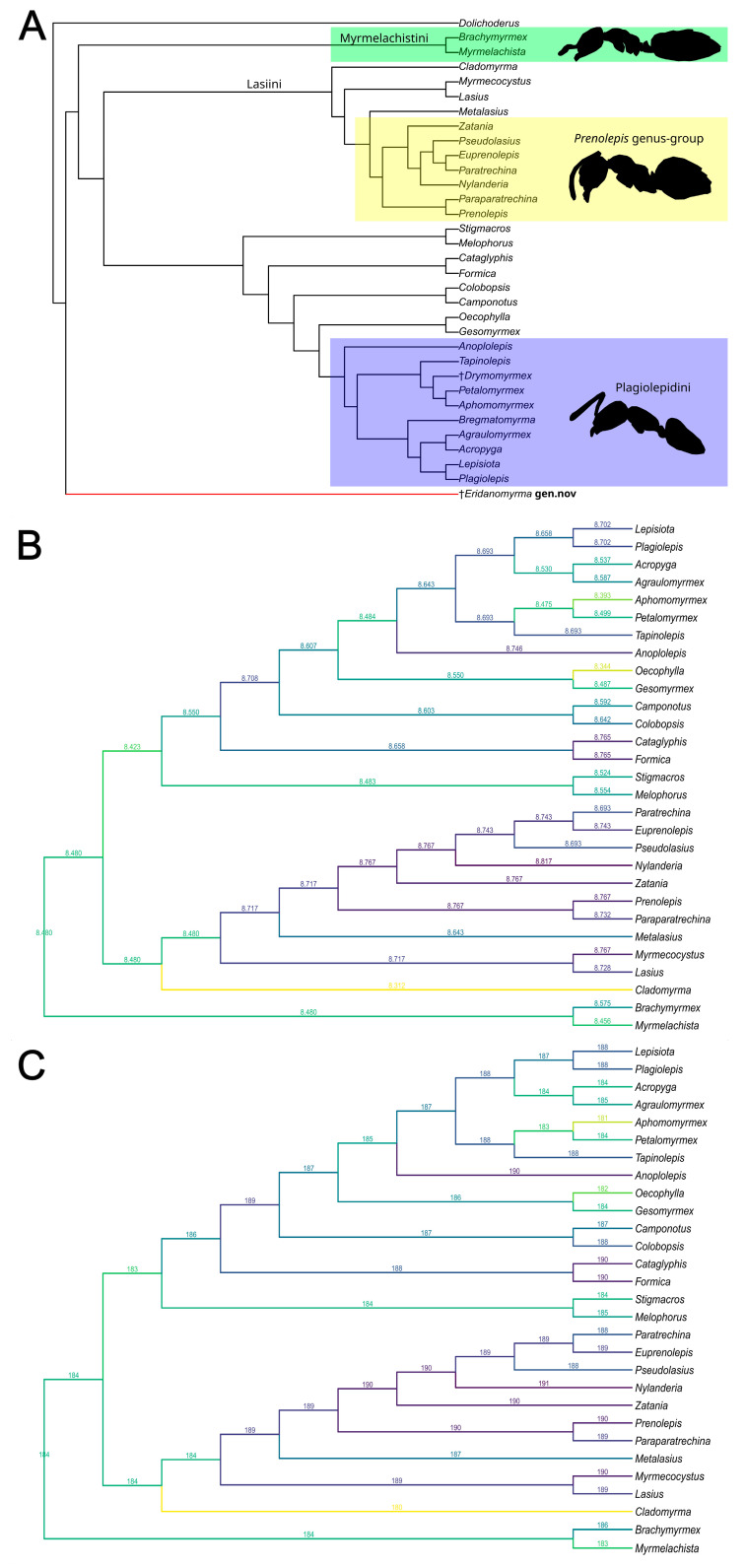
Phylogenetic analyses. (**A**) The most parsimonious placement of †*Eridanomyrma* gen. n., analysed based on the full matrix. Tree resulting from the constrained parsimony analysis under implied weights. (**B**,**C**) Constrained parsimony analyses showing alternative placements of †*Eridanomyrma* gen. n. The score above each branch represents the parsimony score of the topology in which †*Eridanomyrma* gen. n. is inserted to that branch. (**B**) Analysis under equal weights. (**C**) Analysis under implied weights.

In the initial analysis (Figure 3A, Files S1 and S3), all taxa in the morphological matrix were included. For taxa with both morphological and molecular data, their interrelationships were fixed as the backbone tree. The fossil genera (†*Eridanomyrma* gen. n. and †*Drymomyrmex*) and other extant taxa without molecular data (*Bregmatomyrma*) were allowed to move freely across the backbone tree [35,42,43].

The second analysis step (Figure 3B,C, Files S1 and S3) included only Formicinae taxa fixed in the backbone tree and the new fossil genus. Only the new fossil ant was allowed to move freely across the backbone tree. In order to perceive the uncertainty of the fossil placement, the parsimony scores of the trees with alternative placements of the fossil were mapped to the corresponding branches of the backbone tree based on [31,32,33]. The exact phylogenetic placement of †*Eridanomyrma* gen. nov. remains uncertain, and we offer a discussion of its potential affinities based on our constrained phylogenetic analyses. In addition, we have tested the phylogenetic placement of †*Drymomyrmex* and *Bregmatomyrma* (in the absence of molecular data). The taxonomic placement of †*Drymomyrmex* was uncertain, but Radchenko, A.G. [45] suggested that it belonged to the tribe Plagiolepidini, which our analysis confirmed.

## 4. Discussion

The Late Eocene Baltic amber is the largest insect lagerstätte in the world. More than 3500 arthropod species have been described as originating from this amber [46]. Ants are a fairly prominent group, comprising about 5% of all insect remains [47]. At the moment, more than 190 valid species of ants have been described, comprising 22% of all valid fossil species [48]. It is noteworthy that more than 150 years have elapsed since the publication of G. Mayr’s landmark monograph [49], and approximately 20,000 specimens have been examined since that time. This ongoing research has led to the discovery of not only new species, but also new genera of significant interest. According to estimates by Penney and Preziosi [50], approximately 30% of ant species have yet to be discovered in Baltic amber.

The ant †*Eridanomyrma unipetropolitana* sp. nov. has been classified within the subfamily Formicinae by the venation of the forewings, yet it displays a highly specialised morphology and an unconventional array of features that are not observed in extant ant lineages. The most notable characteristic of †*Eridanomyrma* gen. nov. is the distinctive structure of the clypeus, which is characterised by the presence of two outgrowths on the anterior clypeal margin that form distinct, symmetrical, double horns that are pointed in different directions. Such forms of clypeus are not observed among other Formicidae. Some ants of the stem group exhibit a similar morphological syndrome. The cranial horns present in †*Aquilomyrmex*, †*Chonidris* and †*Dhagnathos* are the product of an anterior clypeal margin [10]. However, they possess vertically articulated mouthparts, in which the mandibles interact with the outgrowth of the head capsule.

It has been observed that an adjacent film is present throughout the body of the ant, extending, for instance, to the left eye (Figure 1). The bubbles (in the vicinity of the basal part of the scape, between the forelegs, and near the basal part of the wings, etc.) exhibit a complete merging with the body, both in terms of colour and structure. Consequently, it is not always feasible to ascertain where the true body structure ends and the film begins. It is possible that some seams are obscured by this film, and the lower protruding part of the clypeus between the horns is also covered. The mandibles are also worthy of note, being unusual in form and displaying an external reversion to a sphecoid-like configuration. At the end of the apical teeth on the dorsal surface there is a small file consisting of three small teeth directed distally. We also do not know any queens of the subfamily Formicinae in which the ocelli are completely reduced (although the ocelli may be very reduced and hidden under the film).

Forewing venation generally resembles that of Plagiolepidini, some Lasiini (*Prenolepis* genus group, *Cladomyrma* Wheeler, 1920) and Melophorini (Figure 2D). The mediocubital cell (mcu) is usually absent in representatives of tribes of Plagiolepidini, some Lasiini (*Prenolepis* genus group, *Cladomyrma*), Camponotini, Melophorus Lubbock, 1883, and Oecophyllini. But the forewings of Plagiolepidini and some Lasiini (*Prenolepis* genus-group, *Cladomyrma*) differ in that the length of the 1RS vein is approximately equal to the length of the 1M vein. Together, these veins form an obtuse angle. In Camponotini and Oecophyllini, the veins of 1RS and 1M on the forewing form a 180-degree angle. However, in †*Eridanomyrma* gen. nov. the 5RS vein is almost straight, and its distal margin is attached to 4R without being curved, forming an acute angle. This feature has been observed in the tribe Oecophyllini. In all other taxa listed above, the 5RS vein is attached to 4R with a curved distal margin (at least in all the forewings that have been studied so far). There are no characters that would allow us to place the new genus in any tribe with confidence. Considering these circumstances and the very bizarre morphology of the new ant, we propose considering †*Eridanomyrma* gen. nov. in the new tribe †Eridanomyrmini trib. n.

The general structure (elongated body) is similar to that of female ants living in the stems of living plants, such as *Myrmelachista* Roger, 1863; *Aphomomyrmex* Emery, 1899; *Petalomyrmex* Snelling, 1979; *Cladomyrma* Wheeler, 1920; and *Gesomyrmex* Mayr, 1868. There are several alate females of †*Drymomyrmex* that have been identified in Baltic amber, which also have a similar body structure [45,51]. However, all these ants are characterised by a reduction in antennal segments (usually 7–9 or 10). The †*Eridanomyrma* gen. nov. has a complete set (12). The head with large occipital corners is similar to the *Bregmatomyrma*, which also has 12-merous antennae. However, *Bregmatomyrma* have short bodies and their biology is unknown. The most important characteristic of the ants mentioned above, however, is that they clearly have gnawing mandibles, which are usually used to gnaw holes in the stems of plants. The †*Eridanomyrma* gen. nov. mandibles are completely mysterious, and rather predatory. A similar mandibular structure is found in Crabronidae, Sphecidae and Scoliidae wasps. Such mandibles are necessary for short-term retention of prey for the time necessary to sting it. The main function of retention is performed by the apical teeth, and the basal teeth do not allow the prey to rotate around the axis formed by the apical teeth. In addition, clypeal horns are present in many *Cerceris* s.l., it is assumed that they can use them to compact the walls of the burrow. The presence of similar structures in †*Eridanomyrma* gen. nov. suggests a potential functional analogy, although the precise role of these horns in the fossil taxon cannot be determined with certainty. Although wasps mainly use their front legs for digging, they use these folded mandibles to shovel soil out of the nest. Two-pronged mandibles can also be used to carry relatively large objects, which may involve removing pebbles from a burrow or dragging plant particles to conceal the entrance to a burrow. Representatives of the subfamily of Formicinae, to which the †*Eridanomyrma* gen. nov. belongs, do not have a sting. But the occipital corners of the new ant genus are very large, suggesting significant development of the mandibular muscles. However, the head of new genus is flat and so provides little space for mandible occlusor muscles. Therefore, when hunting, they could simply break the soft integument of their prey with their sharp mandibles, killing it instantly. Mandibles were probably a hunting rather than digging tool, which suggests the ant nested in preexisting hollows. In general, two-toothed mandibles are found in workers of some Dorylinae (e. g. *Dorylus wilverthi* Emery, 1899), in workers of *Leptanilla butelli* Forel, 1913, and in males of some primitive ants (e. g. *Amblyopone australis*, Erichson, 1842). But they look completely different. The elongation of the petiole is rare in females of the Formicinae (it is found in *Cladomyrma*, *Myrmelachista*, *Gesomyrmex*) and favours an increase in muscle for a more mobile gaster. Members of the Formicoidea always have two pairs of intersegmental muscles in the petiole, which fix the position of the gaster and control its movements. Raising and lowering the gaster is the main motor function of the petiolar muscles [52].

**Figure 4:**
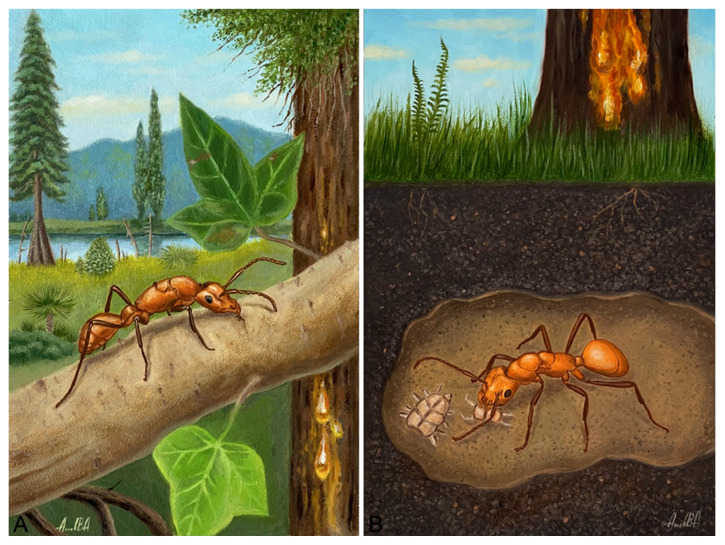
Artistic reconstructions of the supposed lifestyle of †*Eridanomyrma unipetropolitana* sp. nov. (**A**) A dealated female runs along a liana in search of a suitable place for nesting. (**B**) An ant queen feeds on a centipede in an underground nest. Illustrations by Vyacheslav Akaev.

Given all these facts, we assume the following biology of the mysterious extinct ant (Figure 4):

A specialised predator, nesting either in the soil (like *Acropyga* Roger, 1862 and *Agraulomyrmex*, Prins 1983) or in plants (like *Petalomyrmex*, *Cladomyrma*, *Myrmelachista*, etc.) (Figure 4A). Most likely, it had small nests, possibly with trophobionts. Many ants (*Aphomomyrmex*, *Petalomyrmex*, *Cladomyrma*, *Gesomyrmex* and *Tetraponera*) with a similar elongated morphology are often associated with trophobionts (mainly with Hemiptera and Cicadoidea). Trophobiosis has repeatedly developed in ants, most often in the subfamilies Formicinae, Dolichoderinae, and Myrmicinae, and less often in Ponerinae, Ectatomminae, and Pseudomyrmecinae [53]. Trophobiosis is facultative in most ants. However, in very few ant species, trophobiosis is obligate. Some ants (*Agraulomyrmex*, *Acropyga*) associated with trophobionts are hypogeic (living entirely underground). *Acropyga* surviving primarily, it is believed, by “tending” mealybugs (Hemiptera: Pseudococcidae) on underground roots for their “honeydew” [54]. Finally, it cannot be excluded that the new genus is a specialised predator (e.g., on myriapods) with a hidden hypogeic lifestyle (Figure 4B). The reduced ocelli also suggest this. These hypotheses are consistent with the fact that the lifestyle of this new extinct ant was not conducive to its ingestion into the resin, making it the only find in more than 150 years of Baltic amber research.

That the new genus is a social parasite, even an inquiline ant, cannot be ruled out either. Small body size, elongation of the scape, loss of body sculpture, reduced pilosity, reduced palpal segmentation, reduced ocelli, absence of metapleural glands and modification of the mandibles all indicate morphological features that Wilson E. O. described as the “anatomical parasitic syndrome” [55]. The bizarre mandibles of the †*Eridanomyrma* gen. nov. may be an adaptation for killing the host queen. Although many social parasites are phylogenetically close to their host species (Emery’s rule) [56], it is not entirely clear who is the possible host species for †*Eridanomyrma* gen. nov. This makes †*Eridanomyrma* gen. nov. not only a notable morphological anomaly, but also a potential candidate for the earliest fossil evidence of social parasitism in ants. Future discoveries of associated workers and reproductive castes, or even preserved relationships between the host species, may help to more accurately test this hypothesis. At the moment, although definitive evidence of parasitism has not yet been found in the palaeontological record, the totality of morphological features in †*Eridanomyrma* gen. nov. can provide convincing indirect evidence of a socially parasitic lifestyle.

Highly specialised organisms form the basis of the biodiversity of communities, but they are the most vulnerable to any environmental fluctuations. It is likely that †*Eridanomyrma* gen. nov. could not survive the gradual cooling of the climate with the appearance of a more abrupt seasonality that began in the Oligocene [57]. This new taxon highlights the adaptive diversity of a highly specialised, extinct lineage of crown-group ants in the Eocene. Our discovery bridges the gap between stem-group innovations and crown-group diversification, challenging existing paradigms about the adaptative potential of crown ants.

## Data Availability

All the required data relevant to the presented study are included in the manuscript or available via the following link: https://doi.org/10.5281/zenodo.15231627.

## Supplementary Materials

The following supporting information can be downloaded at https://doi.org/10.5281/zenodo.15231627 (accessed on 20 June 2025). File S1: Morphological character matrix; File S2: 3D models of †*Eridanomyrma unipetropolitana* sp. nov.; File S3: script of phylogenetic analysis in R; File S4: CT dataset used to render the 3D reconstructions; File S5: Video of model †*Eridanomyrma unipetropolitana* sp. nov.

## Appendix A

Characters list used in morphological analysis:

All character states were scored as Boolean values, with states defined as TRUE (1) or FALSE (0). Whenever possible, only females were scored; if not, this was specified in the trait. If the trait could not be studied or was too variable, then (?).

Body size proxy.

1. Head width ≥ 1 mm—see [33].
  Compound eyes and ocelli.
2. The middle line of the eye situated at or posterior to head midlength (if false, then situated anterior to head midlength).
3. Eyes are large, about equal to a third of the length of the head or more.
  Mandibles.
4. Mandibular teeth number: (0) greater than 5 teeth; (1) 5 teeth or fewer.
5. Gnawing mandibles (1).
6. At least some mandibular teeth in form of fine serration—Applies primarily to Dolichoderinae.
7. Mandible, third tooth from apex size as large as other teeth (i.e., not reduced)—Bolton [37] inferred that a small third tooth from the apex was probably an ancestral state for the Formicinae, with an enlarged tooth being a synapomorphy for the Camponotini.
8. Mandibular basal and masticatory margins meeting at a strongly oblique angle—Observed among sampled taxa primarily among the *Prenolepis* genus group and some Plagiolepidini, where the juncture between the basal and masticatory margins is clearly defined as an angle.
  Other mouthparts.
9. Palp formula: 6:4 (0); less than 6:4 (1).
  Perioral sclerites.
10. Hypostoma with lateral flanges which are conspicuous in profile view—A defining feature of *Dolichoderus* (see [58]).
11. Pleurostomal (cranial) condyle conspicuously large and rectangular—A newly defined state which is characteristic of the dolichoderomorphs (Aneuretinae + Dolichoderinae).
  Frontoclypeal complex and toruli.
12. Antennal toruli considerably distant from posterior clypeal margin (if FALSE, then closely approximated, abutting, or indenting).
13. Antennal toruli nearly contacting, contacting, or indenting posterior clypeal margin—characterisation of this state drawn from [37].
14. Antennal sockets in full-face view: fully exposed.
15. Clypeus modified for reception of labrum (specifically, clypeus with anterolateral notches; see [33].
  Facial surfaces.
16. Frontal carina present—Frontal carinae are present in the majority of formicoids and, among taxa included in this study, are absent for a few Formicinae.
  States of relative antennomere length.
17. Scape length (SL) ≥ head width (HW).
18. First antennomere bulk (both workers and females).
19. Antennal segment number: 12 segments (0); 11 or fewer segments (1).
20. Antenna filiform or slightly thickened.
21. Antenna gradually incrassate towards apex but without a distinctly differentiated club.
22. Apical antennomeres form a club.
  Antennae
23. Erect macrosetae are present on scape.
  Cranial setation.
24. Coarse, paired macrosetae present on face—This setational pattern is observed in various *Prenolepis* genus group taxa, as well as *Anoplolepis gracilipes*. If it occurs in at least some species. Including the workers.
  Metapleural glands.
25. Metapleural gland: present (1); absent (0)—As with scape-to-flagellum length, presence of the metapleural gland is a canonical defining state of the Formicidae [37,53]. The gland has been variably lost in males and has been lost multiple times in the Camponotini.
  Propodeum.
26. Propodeum armed with spines or other distinct protuberances—Propodeal armature has arisen independently among various Formicidae. Only queens were evaluated.
27. Propodeal spiracle situated at or near posterolateral margin of propodeum (if false, then spiracle situated more anteriorly—Although not maximally consistent, a posteriorly situated spiracle is observed in Camponotini and Lasiini among other taxa.
28. Spiracle on the propodeum is elongate and slit-like (1), (if false spiracle is round (0)).
29. Propodeum produced posterodorsally as shelf overhanging posterior face—observed in *Dolichoderus*, and some *Camponotus*.
30. Propodeum is elongated. (if observed in some species (1), if false (0)). Only queens were evaluated.
  Legs.
31. Metacoxae wideset, with petiolar foramen extending to mesocoxal foramina—One of the defining states of Bolton’s [37] Lasiini tribe group, along with a U-shaped ventral petiolar cross-section.
32. Anterior mesotibial spur/spurs is present.
33. Anterior metatibial spur/spurs is present.
  Abdominal segment II (“petiole”).
34. Petiole strongly inclined anteriorly or with posterior portion elongate—This is a feature of those groups which have the “gaster” overhanging the petiole, including the Tapinomini (Dolichoderinae), Plagiolepidini, and *Prenolepis* genus group.
35. Petiolar node with dorsal armature (spine or spines present).
36. Petiole is elongated with very long, low node. (if observed in some species (1), if false (0)). Only queens were evaluated.
37. Petiole muscle orifice: (0) round; (1) oval. from [38].
  Abdominal segment III.
38. Abdominal segment III tergosternal margins forming narrow shoulder laterad helcium—“Shouldering” of abdominal segment III was used by Bolton [37] to diagnose various groups of the Myrmicinae (e.g., comment iii of the Solenopsidine tribe group), but is also observed in Formicinae, such as the *Prenolepis* genus group and the Plagiolepidini.
39. Abdominal segment III tergosternal margins raised high above helcium—This state, plus the preceding, were used by Bolton [37] to define his Plagiolepidini.
  Metasoma, posterior to segment III.
40. Proventriculus: asepalous (0); sepalous (1).
41. Abdominal terga VI and VII with dense, uniform layer of short and strongly curved setae.
  Wings
42. Forewing of gynes with closed cells 1+2R and 3R, cells rm and mcu are absent.
43. The length of the 1RS vein is approximately equal to the length of the 1M vein.
44. The 1RS vein and the 1M vein together form an obtuse angle. (If TRUE (1), if 1RS vein and 1M vein together form a 180-degree angle (0)).
45. The 2r-rs vein is situated at approximately pterostigma midlength, directed by the lower margin posterodistally.
46. The 4M vein departs from cell 1+2r distally from vein 2r-rs.
47. The 5RS vein is almost straight, its distal margin is attached to 4R without being curved, forming an acute angle.
48. The vein 2r-rs smoothly transitions into the vein 4M, so the vein 4M starts where the vein 2r-rs ends.
49. The length of the cu-a vein is more than 2.5× shorter than 2M + Cu vein.
50. Cell 3R is distinctly narrower than 1+2R.
